# Leiomyoma of the prostate: A case report and literature review

**DOI:** 10.1097/MD.0000000000040340

**Published:** 2024-11-01

**Authors:** Zejun Liu, Ji Lu, Kai Yu, Tengteng Jian, Rui Hu, Min Liu

**Affiliations:** a Department of Urology, The First Hospital of Jilin University, Changchun, Jilin, China.

**Keywords:** case report, prostate leiomyoma, surgery, systematic review, therapeutic options

## Abstract

**Rationale:**

Prostate leiomyoma is a rare condition globally, often challenging to diagnose preoperatively, with most cases being definitively identified through postoperative pathology. This benign tumor generally has a good prognosis and is primarily treated with transurethral resection of the prostate in clinical settings. However, there are no established guidelines or therapeutic protocols for managing this disease.

**Patient concerns:**

The patient was admitted to our hospital’s Department of Urology with complaints of hematuria, urinary frequency, and urgency for 1 month. Pelvic computed tomography revealed an irregular, mass-like high-density shadow posterior to the bladder, indistinctly separated from the prostate. The patient had been diagnosed with renal insufficiency during a physical examination 4 years prior but had not received standardized treatment. Six years ago, the patient underwent electrocision of the prostate at our hospital, and postoperative pathology indicated prostate leiomyoma.

**Diagnoses:**

Postoperative pathology confirmed a diagnosis of prostate leiomyoma.

**Interventions:**

The patient presented with an enlarged prostate and preoperative hematuria. Holmium laser enucleation of the prostate (HoLEP) was performed. The Foley catheter was removed on the second postoperative day, and the patient was discharged 3 days after surgery.

**Outcomes:**

Following discharge, the patient was instructed to undergo reexaminations every 6 months. Current follow-up indicates the patient is in good health, with no recurrence of the mass observed.

**Lessons:**

Prostate leiomyoma is an extremely rare condition, and the current primary approach for managing prostate smooth muscle tumors involves active patient monitoring, regular evaluations, and timely surgical intervention if clinical symptoms emerge. In this study, we present a new case report of prostatic smooth muscle tumor and review the existing literature to explore treatment options for prostate leiomyoma within this field.

## 1. Introduction

Leiomyomas are benign tumors that can develop in any organ, with the uterus being the most common site. The prevalence of leiomyomas in women can reach as high as 70% to 80% by the age of 50, with the highest incidence among women of African descent.^[1]^ Leiomyomas originating in the bladder are rare, representing only 0.05% of all bladder tumors, with approximately 250 cases reported globally, affecting men and women equally. Even fewer leiomyomas originate in the prostate, with fewer than 100 documented cases worldwide to date. Most leiomyomas are diagnosed postoperatively, though they may also present with symptoms of local irritation, such as urinary retention, irritative urethral symptoms, and hematuria. The prognosis for leiomyomas is generally positive, with a low recurrence rate. However, accurately assessing the tumor’s origin, size, and boundaries via ultrasound, CT, or MRI is often challenging. Moreover, no clinical guidelines have been established for diagnosing and managing this condition or for distinguishing it from malignant bladder tumors. In this report, we describe a case of prostate leiomyoma in a 51-year-old male patient. Additionally, we review the available literature on prostate leiomyomas, covering their epidemiology, etiology, clinical presentation, imaging characteristics, and the diagnostic and therapeutic approaches used in the differential diagnosis and treatment of this condition in clinical practice.

## 2. Case description

### 2.1. History and examination

The patient was admitted to the Department of Urology of our hospital with complaints of hematuria, along with urinary frequency and urgency, persisting for 1 month. He had a 5-year history of hypertension, which was well-controlled with oral antihypertensive medication. Four years earlier, he was diagnosed with renal insufficiency but did not receive standardized treatment. Over the past 6 months, his weight has remained stable, and there have been no significant changes. He has also maintained good mental health. Six years ago, the patient underwent transurethral resection of the prostate (TURP) at our hospital due to urinary difficulties. Pathological examination following the surgery confirmed the presence of prostate leiomyoma, and he had a favorable recovery. However, the patient did not follow the recommended 6-month regular checkups, as he experienced a good quality of life without any clinical symptoms.

### 2.2. Imaging findings

**Figure 1.**
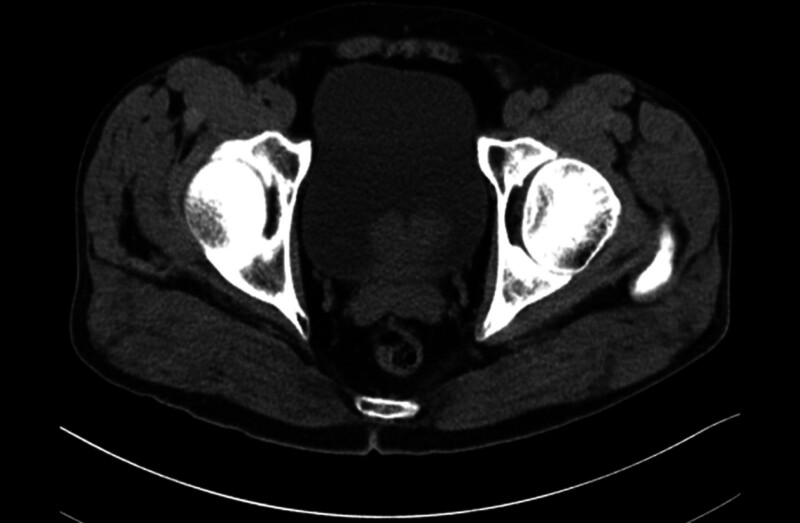
Pelvic CT: an irregular, mass-like high-density shadow is seen in the posterior bladder wall, poorly demarcated from the prostate.

**Figure 2.**
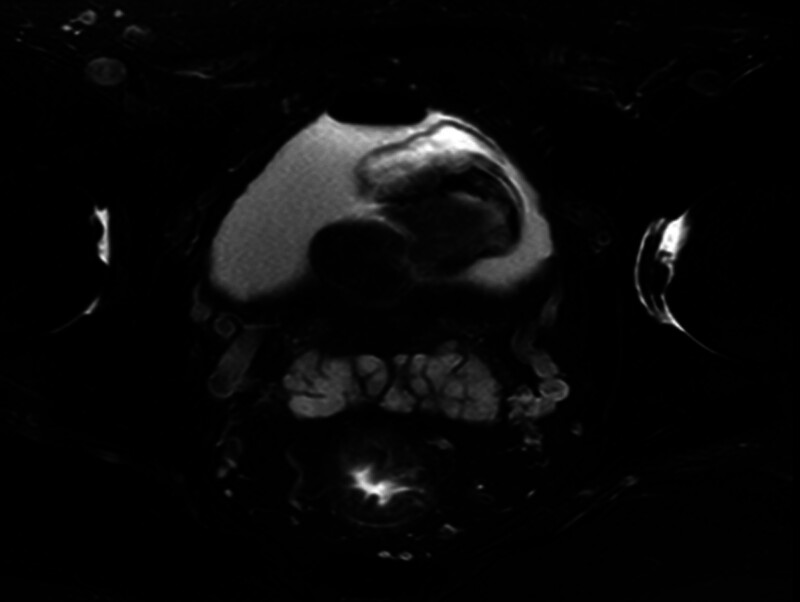
Pelvic MRI: an irregular, mass-like abnormal signal is seen within the bladder cavity.

Pelvic CT revealed a gas shadow within the bladder cavity and an irregular, mass-like high-density shadow in the posterior region of the bladder, which extended into the cavity and was poorly demarcated from the prostate gland. The mass measured approximately 4.2 cm × 3.3 cm × 4.3 cm and contained nodular calcified shadows. Pelvic MRI showed adequate bladder filling, with a mass-like abnormal signal within the lumen, measuring approximately 4.8 cm × 3.8 cm × 3.1 cm. The mass had irregular contours. The T1WI signal was low to slightly high, the T2WI signal was slightly high to slightly low, the DWI signal was high, and the ADC (apparent diffusion coefficient) image was low. The plasma membrane layer appeared smooth, and there was no clear boundary between the lesion and the prostate. The prostate was enlarged, and the central gland showed nodular irregularities in signal intensity, measuring approximately 5.1 cm × 4.5 cm × 2.8 cm. Figures 1 and 2 display the patient’s pelvic computed tomography (CT) and pelvic magnetic resonance imaging (MRI) findings, respectively.

### 2.3. Surgery

**Figure 3.**
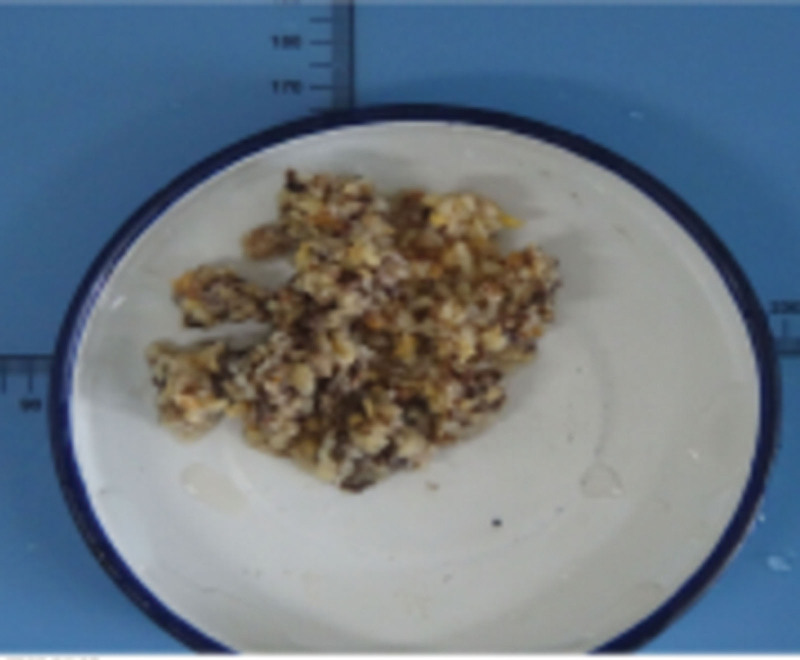
Gross observation of prostate leiomyoma.

Preoperative imaging did not yield a definitive diagnosis of the mass. During the surgery, cystoscopy revealed that the mass originated from the prostate gland and extended from the bladder neck into the bladder. The entire right lobe of the prostate, along with the mass, was successfully removed using holmium laser enucleation of the prostate (HoLEP). Gross anatomical findings are shown in Figure 3.

### 2.4. Histopathological findings

**Figure 4.**
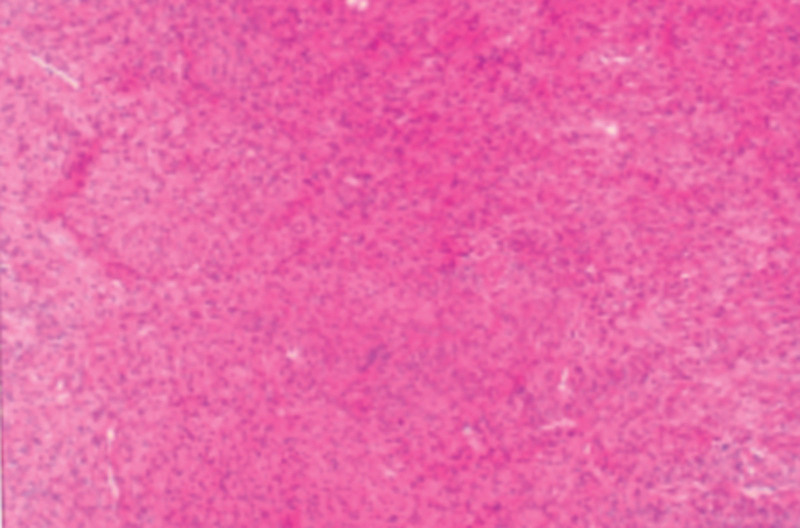
HE-stained section of prostate leiomyoma.

Following a joint diagnosis by 2 senior pathologists at our hospital, the postoperative slow pathology was confirmed. The intraoperative specimen included a prostate measuring 6 cm × 4 cm × 3 cm, along with fragments of light brown crushed tissue. A pathological diagnosis of a mesenchymal-origin tumor with areas of necrosis was made, with regions of increased tumor cell density and some isoforms that were not easily discernible. No nuclear schizophrenia or pathological nuclear schizophrenia was observed. Locally, coated urinary epithelial tissues were visible, and there was no clear anisotropy. These findings are demonstrated in Figure 4. Immunohistochemistry results were as follows: CD34 (−), Desmin (+), Ki67 (+5%), SMA (+), CD117 (−), PR (+).

### 2.5. Postoperative course

**Figure 5.**
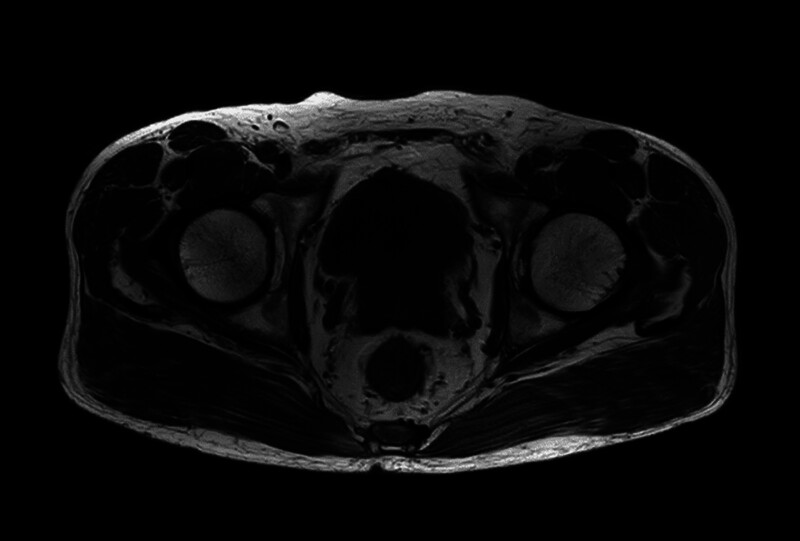
Consistent with postprostate cystectomy changes without recurrence.

The patient showed a good recovery, with the Foley catheter removed on the second postoperative day. He was discharged on the third postoperative day. After discharge, he was advised to undergo periodic reviews of the lesion site every 6 months. At the most recent follow-up, the patient was in good health. A prostate MRI conducted 6 months postoperatively showed no signs of a mass, and the prostate was consistent with the expected postoperative changes. We continue to monitor the patient’s progress. The follow-up prostate MRI is shown in Figure 5.

## 3. Discussion

### 3.1. Epidemiology and etiology

Prostate leiomyoma is a rare condition, with fewer than 100 cases reported in the medical literature globally. Among the documented cases, the disease predominantly affects men aged 50 to 60 years (26%) and 60 to 70 years (41%), while it rarely occurs in younger men under 40 years of age (3%).^[2]^ One case involved a 35-year-old male who was admitted to the hospital with dysuria and an enlarged, firm left-sided prostate leiomyoma. Postoperative pathology confirmed the diagnosis, making him the youngest male patient with a reported prostate leiomyoma worldwide.^[3]^ It is crucial not to overlook the diagnosis of leiomyoma in young patients, even when prostate cancer is suspected. Leiomyomas can develop in any organ containing smooth muscle, including the prostate, bladder, and kidneys within the genitourinary system. However, these benign tumors are more commonly found in the gastrointestinal tract and female genital tract.^[4]^ In 1951, 2 American physicians defined this disease as a prostatic leiomyoma mass measuring 1 cm or more in diameter, with varying amounts of fibrous tissue seen under microscopy, not containing glands, and originating from the prostate.^[5]^ The pathogenesis of prostate leiomyoma remains unclear, but potential causes include long-term chronic inflammation or infection by pathogens. These pathogens may result from repeated irritation from chronic prostatitis or may originate from residual Müllerian duct tissue. The smooth muscle of the prostate and the prostate capsule have been identified as the likely origins of the disease.^[6]^

### 3.2. Clinical presentation

Prostate leiomyoma is a condition that lacks distinct clinical manifestations. The most common symptoms are lower urinary tract symptoms, including urinary frequency, urgency, dysuria, and acute urinary retention.^[7]^ Gross hematuria is rare. Most of the clinical symptoms resemble those of benign prostatic hyperplasia. Patients usually seek medical attention due to lower urinary tract symptoms, such as difficulty in urination, in addition to findings from physical examinations of the prostate mass. Diagnosing prostate leiomyoma based solely on imaging and clinical signs is challenging. However, in cases where the prostate volume exceeds 120 ml or the prostate weight is >100 g, and when clinical symptoms are present, prostate leiomyoma should be considered. A pathological examination is essential for confirming the diagnosis.^[8]^

### 3.3. Diagnosis and radiological characteristic

Prostate leiomyomas are a rare clinical entity, and their diagnosis is not well documented in the literature. Diagnosis primarily depends on pathological examination. Histologically, the disease is characterized by uniformly proliferating spindle cells with well-defined borders. These spindle cells form cross-shaped patterns and contain vesicular nuclei and eosinophilic cytoplasm.^[9]^

The main clinical diagnostic tools include transrectal prostate ultrasound, pelvic CT, prostate MRI, and rectal palpation. However, pathological findings and immunohistochemistry are considered the gold standard for diagnosis. Heterogeneity in tumors is often indicative of a malignant tendency, though prostate leiomyoma shows less pronounced heterogeneity. A review of previous patient samples shows high immunoreactivity for desmin, actin, androgen receptors, and, to a lesser extent, for the wave protein vimentin (vim). Weak CD34 signaling suggests a different direction compared to mesenchymal tumors with malignant potential.^[10]^ Additionally, Ki67 expression is weak in these benign tumors, with most cases showing < 5% expression, indicating a low mitotic rate and a tendency toward benign tumor behavior. In contrast, smooth muscle sarcoma is a more malignant tumor, characterized by increased nuclear pigmentation, nuclear variability, and invasive behavior. Ki67 expression in smooth muscle sarcoma ranges from 20% to 50%,^[11]^ which can help differentiate it pathologically from prostate leiomyoma.^[7]^

CT and MRI can provide a broad assessment of the tumor’s anatomical structure, its size, and its relationship with adjacent tissues. Transrectal prostate ultrasound is 1 of the most basic examinations, though its findings are nonspecific, often showing the mass as hypoechoic or hyperechoic.^[12]^ In some cases, CT scans may reveal a typical round, hypodense mass with smooth, well-defined borders, along with a homogeneously enlarged prostate.^[13]^ Recent literature suggests that the increased use of MRI has significantly improved clinicians’ ability to diagnose and manage benign prostate conditions.^[2]^ On T1-weighted images, a homogeneous mass with an isosignal relative to muscle can be observed, while on T2-weighted images, the mass typically shows a high signal. Additionally, DWI sequences demonstrate limited diffusion with low ADC values. Dynamic contrast-enhanced MRI often shows a nonspecific enhancement pattern. Although the tumor may be large, it usually remains confined and can be clearly differentiated from surrounding normal tissues by its well-defined boundaries.^[14]^ If the imaging pattern appears irregular or if there is an elevated PSA level, a preoperative biopsy should be considered before proceeding with surgery.

### 3.4. Treatment

#### 3.4.1. Operative treatment:

Surgical options for prostate leiomyoma currently include open prostatectomy (ORP), transurethral resection of the prostate (TURP), holmium laser enucleation of the prostate (HoLEP), and prostatic artery embolization (PAE). Before the widespread adoption of endoscopic techniques in the 1990s, most prostate leiomyomas were treated with open resection via the abdomen, with ORP considered the standard approach for this condition.^[15]^

With advancements in technology, minimally invasive surgery has become increasingly common. These techniques allow for tumor removal while preserving the patient’s urinary and sexual functions, minimizing the impact on their quality of life.^[16]^ TURP offers advantages such as shorter hospital stays, significantly less intraoperative blood loss, fewer transfusions, and a lower risk of postoperative complications compared to ORP. However, TURP often requires a longer operative time and careful preoperative assessment of the patient’s overall health.^[17]^ In recent years, HoLEP has proven to be a safe and effective surgical option for prostate tumors of any size. This procedure is associated with shorter operative times and a reduced risk of postoperative urinary incontinence, particularly when there is minimal adhesion between the tumor and surrounding tissues.^[18]^ The critical components of HoLEP surgery include fragmenting and removing the enucleated tissue. In some cases of smooth muscle tumors, even small tumors can present challenges due to their hardness, making fragmentation difficult. In such situations, clinicians may employ “mushroom tactics” using unipolar or bipolar TUR to facilitate tissue fragmentation and its removal through the urethra.^[19]^

Prostatic artery embolization has emerged as a novel treatment option for patients with lower urinary tract symptoms, especially those in poor physical condition or experiencing urinary retention. In 1 study, 2 out of 3 patients who underwent successful embolization demonstrated favorable follow-up imaging results.^[20]^ PAE can be performed under local anesthesia, allowing patients to be discharged the same day. The procedure is relatively simple to learn and is considered safe for patients. However, further research is necessary to assess the long-term efficacy of PAE, as the current literature does not provide clear evidence of its success. Imaging guidance is crucial during the procedure, as the middle rectal artery is often anatomically similar to the prostatic artery, and variations in anatomy may reduce the effectiveness of embolization.^[21]^

#### 3.4.2. Prognosis:

Prostate leiomyoma is generally associated with a favorable prognosis, with atypical stromal hyperplasia and pseudosarcomatous lesions often accompanying this condition. Patients have been followed for extended periods without evidence of recurrence.^[22]^ In Deloar Hossain’s study, it was observed that leiomyomas without mesenchymal overgrowth or transformation into more malignant sarcomas tend to have a positive prognosis, particularly in patients without clinically significant symptoms.^[10]^ Important indicators of prognosis include negative surgical margins and the absence of distant metastatic disease at the time of diagnosis.

## 4. Conclusion

Prostate leiomyoma is a benign tumor, and when imaging reveals a mass in the prostate or bladder with negative PSA or tumor markers, a differential diagnosis of leiomyoma should be considered. Histopathology remains the gold standard for diagnosing this disease, and thorough sampling of postoperative pathology is crucial to rule out significant cellular heterogeneity or necrosis. Most patients undergo surgical treatment and generally experience good outcomes. Currently, minimally invasive surgical approaches are the mainstay of treatment. A variety of surgical options are available, and the choice of treatment must be carefully evaluated by an experienced physician to create an individualized plan. For patients under the age of sixty, preserving sexual function should be considered when devising the surgical strategy. Although the cells in leiomyomas are typically non-heterotypic, if heterotypic features are present, the possibility of malignant transformation should be considered. This paper provides insights into the diagnosis and treatment of prostate leiomyoma, combining available literature and clinical experience. However, due to the limited number of reported cases worldwide and the lack of detailed information for some cases, further clinical research is needed to support these findings.

## Author contributions

**Conceptualization:** Ji Lu, Rui Hu.

**Project administration:** Min Liu.

**Supervision:** Zejun Liu, Tengteng Jian, Min Liu.

**Writing – original draft:** Zejun Liu, Ji Lu, Kai Yu, Tengteng Jian, Rui Hu.

**Writing – original draft:** Zejun Liu, Ji Lu, Kai Yu, Tengteng Jian, Rui Hu.

**Writing – review & editing:** Min Liu.
